# Phytoplankton nutrient dynamics and flow cytometry based population study of a eutrophic wetland habitat in eastern India, a Ramsar site†

**DOI:** 10.1039/c7ra12761h

**Published:** 2018-03-05

**Authors:** Anindita Singha Roy, Prakash Chandra Gorain, Ishita Paul, Sarban Sengupta, Pronoy Kanti Mondal, Ruma Pal

**Affiliations:** Phycology Laboratory, Department of Botany, University of Calcutta 35, Ballygunge Circular Road Kolkata – 700019 West Bengal India rpalcu@rediffmail.com +91-9433116320; Agricultural and Food Engineering Department, Indian Institute of Technology Kharagpur Kharagpur – 721 302 India; Human Genetics Unit, Indian Statistical Institute Kolkata – 700108 West Bengal India

## Abstract

Phytoplankton diversity, their abundance based on flow cytometric (FCM) analysis and seasonal nutrient dynamics were investigated from a waste water fed wetland of Eastern India (88° 24.641′E and 22° 33.115′N). The primary objective of the study was to correlate the seasonal fluctuations in phytoplankton abundance to the environmental variables. Total chlorophyll content and FCM based cell counts were used to characterize and quantify the phytoplankton population. Multivariate statistical methods were employed in predicting the possible relationships between biotic and abiotic variables. Distinct seasonal variations characterized by high abundance during the pre-summer period compared to other seasons were detected. The results indicated that environmental factors like water temperature and nutrients, such as various forms of nitrogen and phosphate, influenced the seasonal phytoplankton accumulation. Cluster analysis and non-metric multidimensional scaling helped analyze the seasonal distribution of phytoplankton based on their composition. The dominant genera among the entire phytoplankton community were *Scenedesmus* spp. of Chlorophyta, followed by *Merismopedia* spp. of Cyanoprokaryota. Around 3.7 × 10^5^ phytoplankton mL^−1^ were recorded during the study period. Due to the very high count of individual species in the community, FCM based counting was applied for determination of Species Diversity Index. The entire population was divided into 13 subpopulations based on the cell sorting method and the seasonal abundance in each sub-population was illustrated.

## Abstract 2

Phytoplankton diversity, their abundance based on flow cytometric (FCM) analysis and seasonal nutrient dynamics were investigated from a waste water fed wetland of Eastern India (88° 24.641′E and 22° 33.115′N).

## Graphical abstract

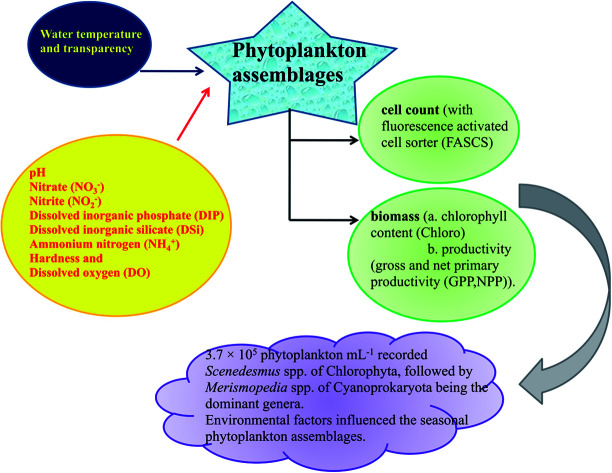

## Introduction

Understanding the various aspects of habitable environments controlling resident community accumulation is a basic research objective in ecology. As phytoplankton are the main organisms responsible for introducing energy into food webs, particular focus has been devoted to understand the factors that control their diversity.^1–3^ The emerging consensus is that biotic–abiotic interactions and ecological drift are the key factors for phytoplankton distribution patterns.^4^ Much research has been centered on open ocean ecosystems where it has long been acknowledged that phytoplankton contribute to ∼50% of global primary production.^1,2^ Their vulnerability to alterations in the physico-chemical parameters of their aquatic environment renders them important biological indicators of water quality.^5,6^ Phytoplankton are potential mitigators of eutrophication since they assimilate excess nutrients rapidly due to their fast growth processes.^5,7–14^ Elevated nutrient levels, especially nitrogen and phosphorus, created by hydrologic processes including water supply, sewage disposal, fisheries, waste water management, recreation, *etc.*, boost phytoplankton populations, leading to extensive blooms.^15^ However, freshwater phytoplankton taxa have N and P requirements different from marine ones, and may respond quite differently to altered nutrient composition.^16^ Even among freshwater taxa, various phylogenetic groups can respond to nutrient conditions in distinct ways.^17,18^

Various methods like microscopic cell counting, chlorophyll estimation, biomass estimation, *etc.* have been exploited for the quantification and characterization of phytoplankton communities as an index of water quality. During the past few decades, flow cytometry (FCM) has been recognized as a potent tool for the study of phytoplankton ecology, especially for studying spatial and seasonal trends.^10,19–21^ Due to the auto-fluorescent properties of the phytoplankton, mixed aquatic populations can be discriminated with the help of FCM.^22^ Generally, allometric and taxonomic analyses of FCM data contribute to characterization of plankton assemblages.^23,24^

Besides open oceans, documentation of standing crop of phytoplankton from different wetlands and their ecological factors have also been carried out throughout the world by various authors in North American Great Plains,^25^ southern coastal areas of North America,^26^ Eastern England,^14^ Eastern Europe,^27^ Southern Africa^28^*etc.* Wetlands are ideal habitats for phytoplankton, which act as nutrient sinks, flood control buffers and breeding grounds for aquatic fauna.^29^ Some noteworthy works on the nutrient dynamics study related to phytoplankton productivity from fresh water wetlands are available.^11,13,30^

In India wetlands are economically important and are mainly used for fish cultivation; moreover, they have distinct architecture, resulting in extensive purification of waste waters.^31^ Pradhan *et al.*,^32^ suggested that phytoplankton growth could be an important factor for greater fish production and could also act as a biomonitor for water quality assessment in the wetland ecosystems. The wetlands of eastern India represent one of the world's largest integrated resource recovery practice based on a combination of aquaculture, agriculture and horticulture practices.

The wetland currently under study is a wastewater fed aquaculture pond of East Kolkata Wetlands (EKW) of Eastern India – a Ramsar site. Here phytoplankton-nutrient dynamics have direct role in fish production together with natural carbon sequestration.^32^ Out of 26 wetlands in India, this wetland is one of the world's largest and oldest integrated resource recovery system based on aquaculture production.^31^ There have been some sporadic reports regarding waste water management at the EKW.^31–35^ The phytoplankton diversity of the EKW has already been reported by some of the present authors.^36–38^ In the present investigation an attempt has been taken to explain the seasonal variations in phytoplankton population in response to changes in environmental variables of EKW with special emphasis on Flow Cytometry based cell sorting methods.

## Materials and methods

### Study area

**Fig. 1:**
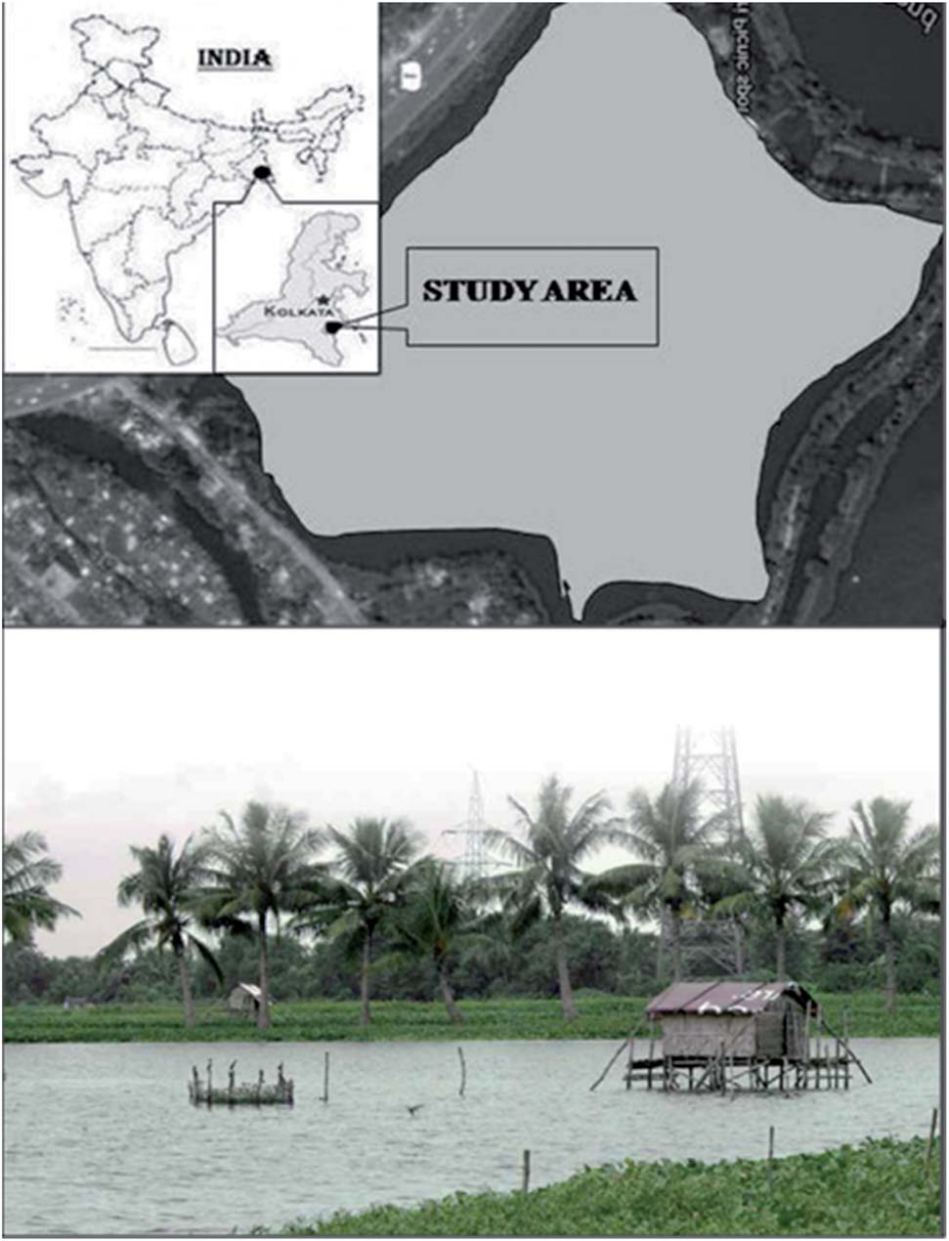
Geographical location and satellite view of the study area.

The East Kolkata Wetlands (EKW) situated along the eastern fringes of Kolkata metropolitan city (West Bengal), make up one of the largest assembly of sewage fed fishponds in the world. It collects sewage wastes from municipalities, agricultural practices and industries of urban and semi-urban areas. It was declared as “Ramsar site number 1203” and based on its ecological and socio-cultural importance, the government of India declared EKW as “Wetland of international importance” under Ramsar convention in the year 2003.^39^ The present study site, called Captain Bhery, was selected within EKW (88° 24.641 ′ E and 22° 33.115 ′ N as determined by Garmin GPS map 76 CSx device) (Fig. 1). It spreads over an area of 125 km^2^ and is1.2–1.5 m deep.^35^

### Water analysis

Samplings for phytoplankton population and water analyses were carried out at bi-weekly intervals for two years from Oct' 13 to Sep' 15, covering different seasons, namely a short post-monsoon (October-November), winter (December-January-February), a pre-summer period (March), a prolonged summer (April-May-June) and monsoon (July-August-September). Sample water was collected in a 1 L PVC (polyvinyl chloride) bottle immersed beneath the surface water from four different transects of the water body. The sample bottles were stored in a cool place and brought back to the laboratory for determination of nutrient concentrations within 30 min of sampling.

The water temperature (temp.) and transparency (transp.) were recorded *in situ* using a Celsius thermometer and a secchi disc respectively. In the laboratory, pH was measured using an electronic pH meter. Different chemical parameters including nitrate (NO_3_^−^) (phenol disulphonic acid method), nitrite (NO_2_^−^) (diazotization method), dissolved inorganic phosphate (DIP) (ammonium molybdate method), dissolved inorganic silicate (DSi) (molybdosilicate method), ammonium nitrogen (NH_4_^+^) (phenate method) and hardness (EDTA titration method) were measured spectrophotometrically following the standard protocols of APHA.^40^

Dissolved oxygen (DO) was measured using Winkler iodometric titration method^41^ using the formula:
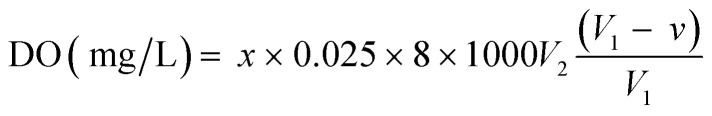
where *V*_1_ = total volume of sample taken (125 mL), *V*_2_ = volume taken for titration (100 mL) *v* = 2 mL (1 mL MnSO_4_ + 1 mL alkaline KI), *x* = volume of sodium thiosulphate consumed in the titration (APHA 1975, 1998).

The gross primary productivity (GPP), net primary productivity (NPP) and community respiration rate (CRR) were determined following light and dark bottle method after 3 hours incubation. Productivity rates were determined by converting DO to carbon equivalence using photosynthetic quotient of 1.2 and respiration quotient of 1.0. Productivity values were determined from the following formulae:
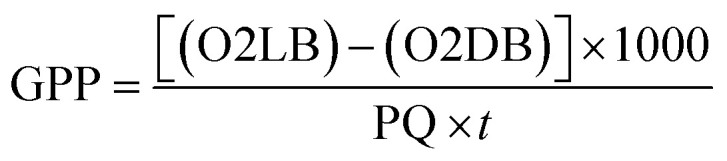

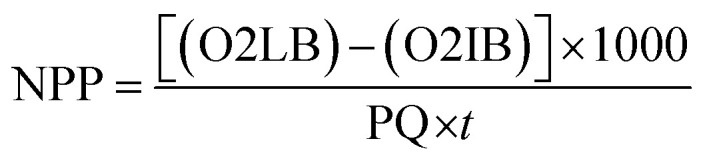

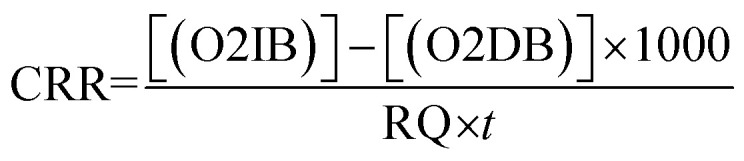
where, O2 LB = DO content of the BOD bottle after incubation in sunlight for 3 hours, O2 IB = DO content of the BOD bottle immediately after sampling, O2 DB = DO content of the BOD bottle after incubation in dark for 3 hours, PQ = photosynthetic quotient(≡ 1.2), *t* = time period of incubation (light/dark) (in hours).

The chlorophyll (chl)concentration was measured spectrophotometrically after extraction in 90% acetone.^42^

### Flow cytometric cell counting and diversity index study

**Fig. 2:**
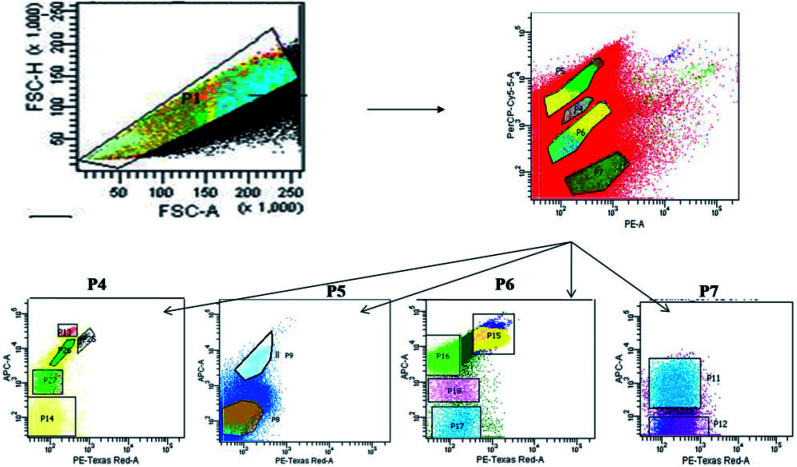
Bivariate scatter plots analyzed using FACSort flow cytometry, showing gating of phytoplankton population based on pigment auto-fluorescence and their cell size.

The sample water was filtered and collected using phytoplankton net (100 μm pore size) to remove unwanted large soil particles, zooplankton or other debris which could block the machine during sample run. The cell count of the natural heterogenous phytoplankton community was carried out with the help of Fluorescence Activated Cell Sorting (FACS) on a BD FACSAria™III cell sorter based on pigment fluorescence, using a method standardized for EKW samples by Roy *et al.*^21^ This FACS machine was equipped with a 100 μm nozzle, with the flow rate of the sample being maintained at 6 μL min^−1^ (lowest possible) and sheath pressure at 20 Psi. Lasers with wavelengths of 375 (ultra-violet), 488 (blue) and 642 (red) nm were used as light source. Optical filters used during the assay included FSC (Filter-488/10), SSC (Filter-488/15), Per CP-CY5-5-A (Filter-695/40), PE (Filter-585/42), PE-Texas Red (Filter-616/23) and APC (Filter-660/20). All parameters were adjusted to logarithmic scale. The choice of gating parameters was based on phytoplankton cell size and their pigment autofluorescence properties. The time and number of events occurring were recorded for estimating the cell concentration. The number of events occurring for each sample corresponded to number of cells passing through the detector. Forward scatter (FSC) filters indicated the cell size and shape while the side scatter (SSC) filters indicated cell granularity, size and refractive index by measuring scattering of incident radiation at 488 nm. Two FSC filters were used to channel and record events corresponding to individual cells within a size limit. The recorded unicelled population for each sample was produced as a cytogram represented as population 1 (P1) (Fig. 2).^21^

The optical filters other than FSC and SSC transmitted autofluorescence wavelengths emitted by different major photo-pigments excited by lasers. For instance, chlorophyll-a on excitation at 488 nm, emits red fluorescence at around 685 nm which can be transmitted through PE-Texas Red (Filter-616/23) filter. Again, phycoerythrin on excitation at 496 nm, emits orange fluorescence at 560–585 nm transmitted through PE (Filter-585/42) filter.^43,44^ Similarly, another important photo-pigment, phycocyanin, emits blue-green fluorescence at around 670 nm on being excited at 650 nm, transmitted through APC (Filter-660/20) or PE-Texas Red (Filter-616/23) filters.^45^ Red autofluorescence (at 695 nm) of peridinine–chlorophyll–protein complex within photosynthetic apparatus excited by 482 nm radiation was transmitted through Per CP-CY5-5-A (695/40) filters.^46^ Each P1 was gated and sorted into smaller entities (P4, P5, P6 and P7) based on two-colour pigment fluorescence (Fig. 2).^21^ Sorted entities (P4–P7) were subdivided on the basis of two-colour pigment fluorescence using a different set of filters, so that 13 distinct and consistent sub-populations (P8, P9, P11–P18, P25–P27) were obtained. These sub-populations were studied for a 24 month period (October 2013–September 2015) to find out seasonal variation patterns among the phytoplankton assemblages.

The gating of the entire population into subpopulations was followed by cell sorting of those gated populations in 4 way sorting precision. After cell sorting each sub-population (P8, P9, P11–P18, P25–P27) was collected on a microplate. The microplate with the sorted samples was identified under light microscope (BD Pathway 855), showing the different phytoplankton taxa obtained. Flow cytometric determination of abundance of each phytoplankton sub-population in each sample was estimated using the formula: *N* = (*n* × 1000) (*q* × *t*)^−1^, where *q* is the flow rate (μL min^−1^), *t* is the duration of analysis, *n* is the number of events counted by the FACS, and *N* is the number of cells per milliliter.

The diversity index of the phytoplankton community study was determined using Shannon Wiener's Index (H′), species richness and species evenness (e).^47^

### Statistical analysis

The statistical analysis was performed in the free and open-source statistical software R version 3.2 (R Core Team 2014). Datasets included environmental variables (pH, temp., transp., DO, BOD, GPP, NPP, CRR, NO_3_^−^, NO_2_^−^, NH_4_^+^, DIN, DSi, DIP, hardness) against a biotic variable (total chl content). Multivariate analyses such as principal component analysis (PCA) and cluster analysis (CA) were performed to envisage the possible relationships between biotic and abiotic variables and to group these variables based on similarity. For the multivariate analysis, excessively rare species (below 1% abundance) were removed from the original dataset as the inclusion of very rare species weakens correlation analysis.^48^ The correlation structure within the set of environmental variables was visualized by plotting the correlations of the variables against PCA axes. Since each principal component (PC) is a linear function of the given variables, the loadings represent the correlations of the variables to PC1 and PC2. For CA, unweighted pair group average (UPGA) method was followed for better understanding of species succession where seasonal assemblages were separated according to the similarity of species composition. However, the level of abundance among the phytoplankton taxa fluctuated according to seasonal variation. Thus in order to get rid of biasness, we scaled the data before clustering by subtracting with the mean followed by dividing with standard deviation. The new scaled data was then used to draw the dendogram and other plots. In general, the goal of the analysis is to detect meaningful underlying dimensions to explain observed similarities or dissimilarities between the investigated objects.

## Results

### Physicochemical parameter analysis

**Fig. 3:**
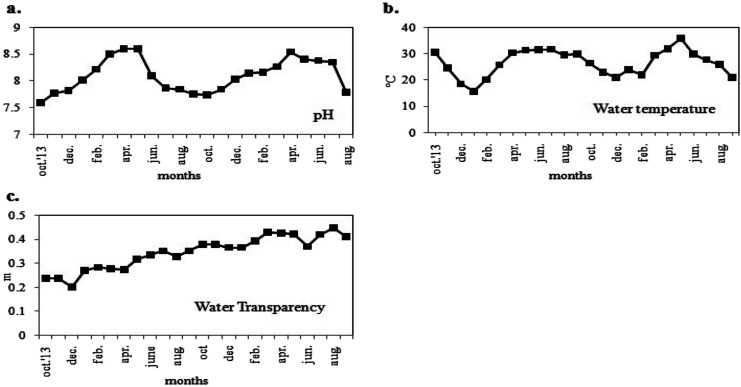
Variations in monthly average values of physical parameters (a) pH, (b) water temperature and (c) water transparency of the study area.

**Fig. 4:**
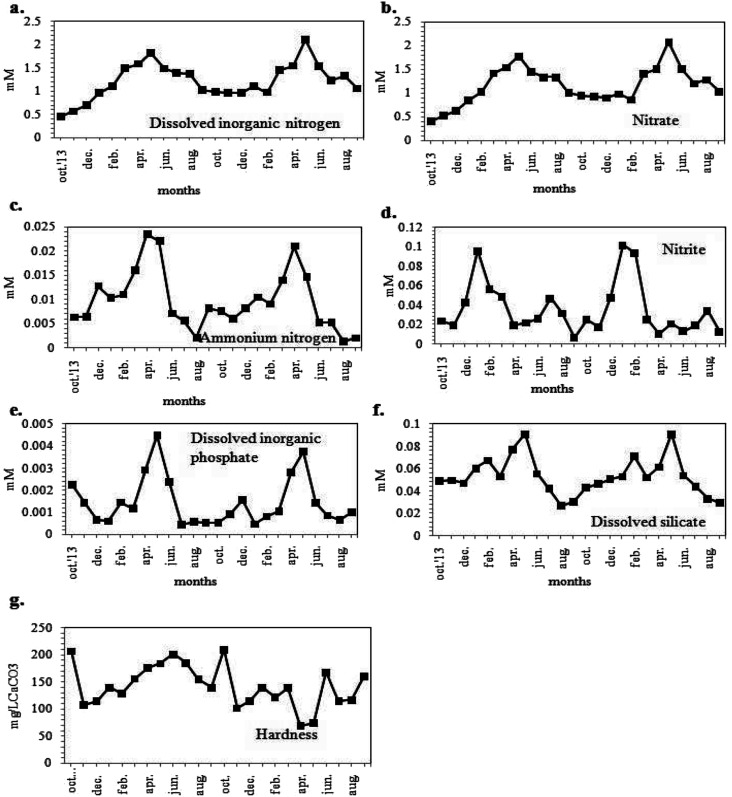
Variations in monthly average values of chemical parameters (a) dissolved inorganic nitrogen (DIN), (b) nitrate (NO_3_^−^), (c) ammonium nitrogen (NH_4_^+^–N), (d) nitrite (NO_2_^−^), (e) dissolved inorganic phosphate (DIP), (f) dissolved silicate (Dsi) and (g) hardness.

The two-year long study investigated the seasonal variations in physicochemical parameters and phytoplankton productivity in Captain Bhery (Fig. 3 and 4). The average values of each parameter recorded as two datasets at fifteen days intervals in each month were taken into consideration. The water was found to be alkaline with pH values ranging from 7.6 to 8.6 units (Fig. 3a). Minimum pH values were recorded during post-monsoon seasons, while maximum pH was obtained during summer for both years. The water temperature followed a seasonal cycle with a maximum mid-summer value at 36 °C and a minimum in mid-winter value at 15.7 °C (Fig. 3b). The water transparency remained almost same with minute fluctuations between 0.20 m and 0.447 m throughout the year (Fig. 3c).

The dissolved inorganic nitrogen (DIN) concentration of the habitat water (Fig. 4a) varied from 441.80 to 2112.03 μM. The DIN was found to occur in natural waters in various forms, including NO_3_^−^, NO_2_^−^ and NH_4_^+^ with NO_3_^−^ as the most common form. The values of NO_3_^−^, NH_4_^+^ and NO_2_^−^ contents of the sample water ranged from 411.45 to 2076.49 μM, 1.37 to 23.67 μM and 6.52 to 101.74 μM respectively, with maximum concentrations during the summer season (Fig. 4b–d). The DIP concentration obtained was maximum in May 2012 (4.47 μM) and minimum during the winters (0.45 μM) (Fig. 4e). The concentrations of DIN and DIP validated the eutrophic status of the water body.^49^ It was apparent from Fig. 4f that the DSi concentration ranged from 29.42 to 91.18 μM showing maxima in summer season. The sample water was hard, with hardness values ranging between 69.00 to 210.00 mg CaCO_3_ L^−1^ (Fig. 4g).

**Fig. 5:**
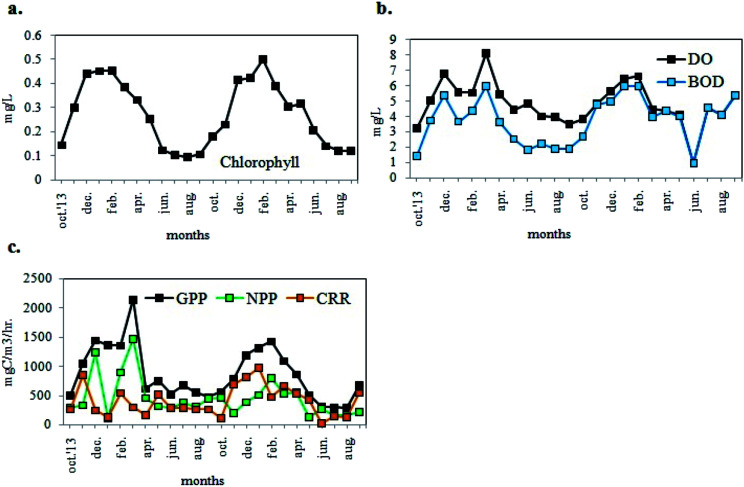
Variations in monthly average values of (a) chlorophyll, (b) DO and BOD, (c) GPP, NPP, CRR of the experimental site.

Phytoplankton biomass was estimated in terms of chl (chlorophyll) content. The chl content of sampled water ranged from 0.095 to 0.501 mg L^−1^ (Fig. 5a) with values higher during the pre-summer and lower during the post-monsoon for both years. In addition to the estimation of the phytoplankton abundance, their photosynthetic activity was also determined in terms of DO. The DO level of the sample water varied from 3.24 mg L^−1^ to 8.14 mg L^−1^ following similar trend in seasonal variation as that of chl content (Fig. 5b), and showing maximum values in winter. The BOD value ranged from 1.44 to 6.0 mg L^−1^ (Fig. 5b). As evident from Fig. 5c, the values of GPP, NPP and CRR ranged from 283.61 to 2147.71 mgC m^−3^ h^−1^, from 114.11 to 1471.86 mgC m^−3^ h^−1^ and from 26.00 to 976.67 mgC m^−3^ h^−1^ respectively. The GPP values were higher than NPP, thereby establishing a positively productive ecosystem (Fig. 5c). Maximum productivity (GPP) was recorded in the month of March 2014 and minimum productivity was recorded in August 2015.

Correlation matrix (Table 1) based on Pearsonian *r* values (*N* = 48) revealed that chl had significant negative correlation with temp (*r* = −0.644) and hardness (*r* = −0.359); while very weak to moderately negative correlations with pH (*r* = −0.018), NO_3_^−^ (*r* = −0.148), DIN (*r* = −0.115). However, significant positive correlations were obtained between chl and NH_4_^+^ (*r* = 0.489), NO_2_^−^ (*r* = 0.627), Dsi (*r* = 0.511), while with DIP (*r* = 0.029) a weak positive correlation was obtained.

**Table 1:** Correlation matrix of chlorophyll content of phytoplankton biomass *vs.* environmental variables

	Chl		pH	Temp	Transp	DO	BOD	GPP	NPP	CRR	NO_3_^−^	NO_2_^−^	NH_4_^+^	DIN	Dsi	DIP	Hardness
Chl	1	−0.018		**−0.644** a	−0.237	**0.713** a	**0.623** a	**0.474** a	**0.536** a	**0.419** a	−0.148	**0.627**	**0.48**9^a^	−0.115	**0.511** a	0.029	**−0.359** a
pH	−0.018	1		**0.415** a	**0.356** a	0.019	−0.055	0.102	−0.054	−0.196	**0.780** a	−0.092	**0.354** a	**0.786** a	**0.501** a	**0.499** a	−0.022
Temp	−**0.644**^a^	**0.415** a		1	**0.357** a	**−0.605** a	−**0.522**^a^	−0.243	−**0.352**^a^	−0.237	**0.625** a	−**0.707**^a^	0.106	**0.595** a	0.098	**0.519** a	0.187
Transp	−0.237	**0.356** a		**0.357** a	1	−0.278	0.138	−**0.316**^a^	−**0.389**^a^	0.094	**0.448** a	−0.134	−0.182	**0.445** a	−0.095	−0.040	**−0.304** a
DO	**0.713** a	0.019		**−0.605** a	−0.278	1	**0.791** a	**0.514** a	**0.711** a	**0.299** a	−0.180	**0.567** a	0.279	−0.151	0.199	−0.158	−0.171
BOD	**0.623** a	−0.055		**−0.522** a	0.138	**0.791** a	1	**0.333** a	**0.456** a	**0.489** a	−0.176	**0.453** a	0.170	−0.154	0.104	−0.208	**−0.538** a
GPP	**0.474** a	0.102		−0.243	**−0.316** a	**0.514** a	**0.333** a	1	**0.402** a	0.088	−0.022	0.252	**0.372** a	−0.008	0.172	0.166	−0.037
NPP	**0.536** a	−0.054		**−0.352** a	**−0.389** a	**0.711** a	**0.456** a	**0.402** a	1	0.052	−0.147	0.273	**0.351** a	−0.133	0.077	−0.160	−0.035
CRR	**0.419** a	−0.196		−0.237	0.094	**0.299** a	**0.489** a	0.088	0.052	1	−0.190	0.200	0.115	−0.181	0.138	0.071	**−0.383** a
NO_3_^-^	−0.148	**0.780** a		**0.625** a	**0.448** a	−0.180	−0.176	−0.022	−0.147	−0.190	1	−**0.287**^a^	**0.418** a	**0.999** a	**0.445** a	**0.541** a	−0.058
NO_2_^-^	**0.627** a	−0.092		**−0.707** a	−0.134	**0.567** a	**0.453** a	0.252	0.273	0.200	−**0.287**^a^	1	0.004	−0.239	0.137	−**0.327**^a^	−0.036
NH_4_^+^	**0.489** a	**0.354** a		0.106	−0.182	0.279	0.170	**0.372** a	**0.351** a	0.115	**0.418** a	0.004	1	**0.428** a	**0.724** a	**0.597** a	−0.075
DIN	−0.115	**0.786** a		**0.595** a	**0.445** a	−0.151	−0.154	−0.008	−0.133	−0.181	**0.999** a	−0.239	**0.428** a	1	**0.461** a	**0.533** a	−0.061
Dsi	**0.511** a	**0.501** a		0.098	−0.095	0.199	0.104	0.172	0.077	0.138	**0.445** a	0.137	**0.724** a	**0.461** a	1	**0.691** a	−0.083
DIP	0.029	**0.499** a		**0.519** a	−0.040	−0.158	−0.208	0.166	−0.160	0.071	**0.541** a	**-0.327** a	**0.597** a	**0.533** a	**0.691** a	1	−0.004
Hardness	**−0.359** a	−0.022		0.187	**−0.304** a	−0.171	**−0.538** a	−0.037	−0.035	−**0.383**^a^	−0.058	−0.036	−0.075	−0.061	−0.083	−0.004	1

a Correlation is significant at the 0.05 level (2-tailed), *r*= Pearson correlation, *N* = 48.

The DO and photosynthetic productivity (GPP and NPP) showed significant positive correlations with chl (Table 1). A significant positive correlation of DO with GPP (*r* = 0.514) suggested an increased oxygen concentration with higher photosynthetic activity. However, increased BOD levels were observed with increasing values of chl, DO and GPP. The DO values also showed negative correlation with temp. (*r* = −0.605), probably indicating inverse relationship between the solubility of oxygen in water and temperature.

Different nutrient parameters like NO_3_^−^, NH_4_^+^, DIN, DIP, Dsi and hardness of the habitat water showed significant positive correlations with the pH of the water (Table 1), thereby indicating their contribution towards the alkaline nature of the habitat water. Again, NO_3_^−^, DIN and DIP were significantly correlated with temp., suggesting a probable higher nutrient concentration during the warm season.

### Phytoplankton community study

**Fig. 6:**
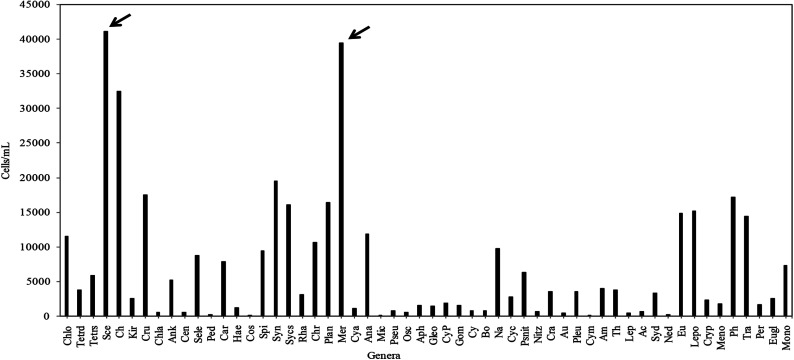
Total cell count of the different phytoplankton genera recorded by FACS study (full forms of the abbreviated names are listed). Ch: *Chlorella* sp., Tetrd: *Tetraedron* sp., Tetrs: *Tetrastrum* sp., Sce: *Scenedesmus* sp., Chlo: *Chlorococcum* sp., Kir: *Kirchneriella* sp., Cru: *Crucigenia* sp., Chla: *Chlamydomonas* sp., Ank: *Ankistrodesmus* sp., Cen: *Centritactus* sp., Sele: *Selenastrum* sp., Ped: *Pediastrum* sp., Car: *Carteria* sp., Hae: *Haematococcus* sp., Spi: *Spirulina* sp., Syn: *Synechococcus* sp., Sycs: *Synechocystis* sp., Rha: *Rhabdoderma* sp., Chr: *Chroococcus* sp., Plan: *Planktolyngbya* sp., Mer: *Merismopedia* sp., Cya: *Cyanarcus* sp., Ana: *Anabaenopsis* sp., Mic: *Microcystis* sp., Pseu: *Pseudoanabaena* sp., Osc: *Oscillatoria* sp., Aph: *Aphanocapsa* sp., Gleo: *Gleocyctis* sp., Gom: *Gomphosphaeria* sp., Cy: *Cylindrospermopsis* sp., Bo: *Borzia* sp., CyP: Cyanophytic population, Na: *Navicula* sp., Cyc: *Cyclotella* sp., Psnit: *Pseudonitzschia* sp., Nitz: *Nitzschia* sp., Cra: *Craticula* sp., Ned: *Nedium* sp., Eu: *Euglena* sp., Lepo: *Lepocinclis* sp., Cryp: *Cryptoglena* sp., Mono: *Monomorphina* sp., Ph: *Phacus* sp., Tra: *Trachelomonas* sp., Per: *Peranemopsis* sp., Eugl: *Euglenaria* sp., Meno: *Menodinium* sp., Cos: *Cosmarium* sp.).

The phytoplankton community study from the investigated wetland revealed the presence of diverse groups of unicellular or colonial, free-floating autotrophic microplanktonic taxa belonging to mainly four different algal phyla, namely Cyanoprokaryota, Chlorophyta, Bacillariophyta and Euglenophyta. Among the total 165 taxa recorded (Table 2), the chlorophytic members were found to account for the bulk phytoplankton population with 84 species followed by Cyanoprokayotes (30 species), Euglenophytes (28 species) and Bacillariophytes (23 species) (Table 2). Taxonomic identification of the above recorded taxa had been previously carried out.^36–38^ In the EKW a total of 3.7 × 10^5^ phytoplankton per mL was recorded during the entire study period, where pre-summer season was the most productive (9.2 × 10 ^4^ cells per mL) and post monsoon was the least (6.5 × 10^4^ cells per mL). From the total cell count of individual phytoplankton genera, it was also evident that *Scenedesmus* sp. from phylum Chlorophyta followed by *Merismopedia* sp. of Cyanoprokaryotes showed the maximum profusion (Fig. 6).

**Table 2:** The floristic list of phytoplankton taxa recorded during the study period from East Kolkata Wetlands

Chlorophyta (51%)		Cyanoprokaryota (18%)	Bacillariophyta (17%)	Euglenophyta (14%)
*Chloroccum humicola*	*Desmodesmus bicaudatus*	*Merismopedia minima*	*Aulacosiera granulata*	*Euglena viridis*
*C. echinozygotum*	*D. pleiomorphus*	*M. punctata*	*Navicula phyllepta*	*E. polymorpha*
*Chlamydomonas mucicola*	*D. itascaensis*	*M. trolleri*	*N. cryptocephala*	*E. tuberculata*
*C. globosa*	*D. quadricauda*	*M. glauca*	*N. tripunctata*	*E. gracilis*
*Stauridium tetras*	*D. armatus* var. *bicaudatus*	*Planktolyngbya contorta*	*N. peregrine*	*E. deses*
*S. tetras* var. *apiculatum*	*D. abundans*	*Anabaenopsis circularis*	*N. pupula*	*E. acus*
*Pediastrum privum*	*D. opoliensis*	*A. tanganyikae*	*Cocconeis pediculus*	*Euglenaformis proxima*
*P. boryanum*var. *brevicorne*	*D. quadricauda* var. *longispinum*	*A. arnoldii*	*C. costata*	*Lepocinclis globulus*
*P. duplex* var. *clathratum*	*Tetrastrum triangulare*	*A. raciborskii*	*Cymbella lanceolata*	*L. ovum*
*P. boryanum* var. *perforatum*	*T. heteracanthum*	*Chroococcus limneticus*	*Cyclotella striata*	*L. salina*
*P. duplex* var. *duplex*	*T. staurogeniaeforme*	*C. dispersus*	*C. meneghiniana*	*L. salina* var. *vallicauda*
*P. subgranulatum*	*Treubaria setigera*	*C. dispersus* var. *minor*	*Pseudonitzschia sp.*	*Monomorphina pseudonordstedii*
*P. sarmae*	*Schroederia judayi*	*C. turgidus*	*Craticula halophila*	*Trachelomonas volvonica*
*P. duplex* var. *genuinum*	*Eutetramorus tetrasporus*	*Synechococcus elongatus*	*C. cuspidata*	*T. volzii* var. *intermedia*
*Lacunastrum sp.*	*Monoraphidium minutum*	*Synechocystis aquatilis*	*Nitzschia acicularis*	*T. intermedia*
*Pseudopediastrum boryanum*	*M. contortum*	*Spirulina subsalsa*	*N. palea*	*Cryptoglena skujae*
*Tetraedron minimum*	*Crucigenia quadrata*	*S. subtilissima*	*N. frustulum*	*Peranemopsis trichophora*
*T. muticum*	*C. tetrapedia*	*S. laxissima*	*N. fruticosa*	*Phacus tortus*
*T. caudatum*	*Chlorella vulgaris*	*S. nordstedtii*	*Pleurosigma angulatum*	*P. acuminatus*
*T. caudatum* var. *longispinum*	*C. ellipsoidea*	*Oscillatoria acutissima*	*Amphora coffaeformis*	*P. caudatus*
*T. trigonum*	*Crucigeniella crucifera*	*O. rubescens*	*Thalassiosira weissflogii*	*P. curvicauda*
*T. trigonum* var. *gracile*	*C. apiculata*	*Rhabdoderma irregulare*	*Leptocylindricus danicus*	*P. anacoleus* var. *undulatus*
*T. regulare*	*C. rectangularis*	*R. lineare*	*Acnanthes sp.*	*P. glaber*
*T. pusillum*	*C. irregularis*	*Coelosphaerium palladium*	*Synedra ulna*	*P. chloroplastes* var. *incisa*
*Scenedesmus dimorphus*	*Ankistrodesmus gracilis*	*Gomphosphaeria aponina*		*P. sesquitortus*
*S. denticulatus*	*A. falcatus*	*Rhabdogloea fascicularis*		*P. longicauda*
*S. bernardii*	*A. falcatus* var. *acicularis*	*R. raphidioides*		*Rhabdomonas costata*
*S. acuminatus*	*A. falcatus* var. *tumidus*	*Pseudoanabaena catenata*		*Euglenaria sp.*
*S. ecornis*	*A. falcatus* var. *stipitatus*	*P. galeata*		
*S. acutus*	*A. convolutus*	*Microcystis aeruginosa*		
*S. bijuga*	*Selenastrum bibraianum*			
*S. pleiomorphus*	*S. gracile*			
*S. disciformis*	*S. westii*			
*S. pseudoopliensis*	*Actinastrum gracillum*			
*Coelastrum microporum*	*Mucidosphaerium sphagnale*			
*C. reticulatum*	*Mucidosphaerium sp.*			
*C. proboscidium*	*Desmococcus olivaceum*			
*C. pseudomicroporum*	*Carteria cerasiformis*			
*Kirchneriella lunaris*	*Hematococcus lacustris*			
*K. contorta*	*Deasonia granata*			
*K. obese*	*Oocystidium ovale*			
*K. elongata*	*Oocystis borgei*			

**Fig. 7:**
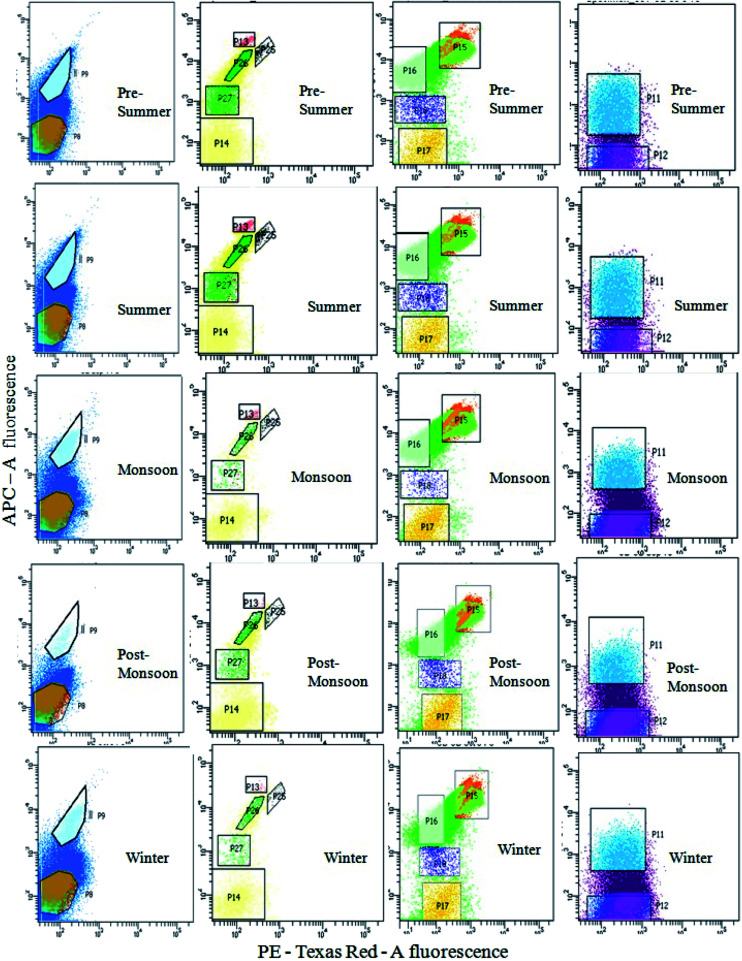
APC-A fluorescence *vs.* PE-Texas Red-A fluorescence cytograms showing the seasonal variations in phytoplankton population at different seasons. From left to right, cytograms in each row represent P5, P4, P6 and P7, respectively.

Seasonal distribution of the phytoplankton community composition was revealed from the FACS study. Populations of cells (P4–P7) sorted on the basis of two-color pigment fluorescence intensity at two FACS filters were further sorted based on fluorescence intensity at two other filters (Fig. 2). The phytoplankton abundance in terms of cell count was mapped based on these sorted sub-populations (P8, P9, P11– P18, P25– P27) representing mixed assemblages of taxa with similar pigment profiles (Table 3), and plotted on cytograms for different seasons (pre-summer, summer, monsoon, post-monsoon and winter) (Fig. 7). Each of these subpopulations was tagged by a specific colour (Table 3). The change in colour intensity of the subpopulations during different seasons indicated their variations in abundance at different seasons in terms of abundance. Colour intensity of any one sub-population was directly proportional to number of cells in the corresponding assemblage (Fig. 7). In general, comparatively higher cell counts were obtained for all assemblages during the pre-summer, while lower counts occurred in post-monsoon (for P11, P12, P8 and P9), monsoon (for P15–P18) and winter (for P13, P14, P25–P27). Along with the phytoplankton abundance, types of phytoplankton recorded from each population were also studied through microscopic identification (Table 3). Most of the sub-populations obtained by FACS consisted of mixtures of microplanktonic phyla, although a few (P14, P17, P25 and P26) contained members of single phyla only.

**Table 3:** List of observed genera belonging to different phylum as recorded from FACS study

Sorted population (distinguished by different colours)	Phytoplankton diversity observed through microscopic analysis
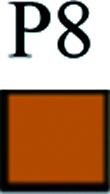	Chlorophytes (*Tetrastrum* sp., *Scenedesmus* sp., *Chlorella* sp., *Tetraedron* sp., *Chlorococcum* sp., *Kirchneriella* sp., *Ankistrodesmus* sp., *Crucigenia* sp., *Selenastrum* sp., *Pediastrum* sp., *Selenastrum* sp.), Cyanoprokaryotes (*Spirulina* sp., *Synechocystis* sp., *Rhabdoderma* sp., *Merismopedia* sp.)
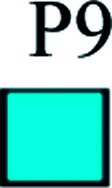	Chlorophytes (*Chlorococcum* sp., *Tetrastrum* sp., *Scenedesmus* sp., *Chlorella* sp., *Kirchneriella* sp., *Ankistrodesmus* sp., *Selenastrum* sp., *Eutretramorus* sp. *Monoraphidium* sp.), Euglenophytes (*Lepocinclis* sp., *Euglena* sp., *Phacus* sp.)
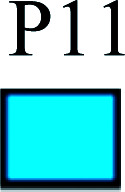	Chlorophytes (*Chlorococcum* sp., *Tetrastrum* sp., *Scenedesmus* sp., *Chlorella* sp., *Kirchneriella* sp., *Tetraedron* sp., *Crucigenia* sp., *Selenastrum* sp., *Pediastrum* sp.), Bacillariophytes (*Navicula* sp., *Nitzschia* sp., *Pseudonitzschia* sp., *Craticula* sp., *Pleurosigma* sp.)
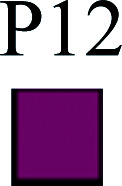	Cyanoprokaryotes (*Spirulina* sp., *Merismopedia* sp., *Rhabdoderma* sp.), Euglenophytes (*Trachelomonas* sp.), Bacillariophytes (*Cyclotella* sp., *Cocconies* sp., *Amphora* sp., *Thalassiosira* sp.)
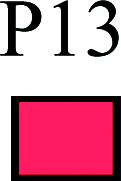	Chlorophytes (*Scenedesmus* sp.), Euglenophytes (*Trachelomonas* sp., *Euglenaria* sp.), Bacillariophytes (*Aulacosiera* sp., *Leptocylindricus* sp., *Acnanthes* sp*.*, *Synedra* sp.)
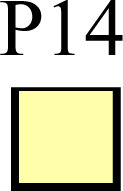	Cyanoprokaryotes (*Rhabdoderma* sp., *Rhabdogloea* sp.)
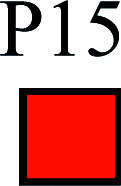	Chlorophytes (*Kirchneriella* sp., *Crucigenia* sp., *Chlamydomonas* sp.), Cyanoprokaryotes (*Spirulina* sp., *Synechococcus* sp., *Synechocystis* sp., *Chroococcus* sp., *Planktolyngbya* sp., *Merismopedia* sp., *Pseudoanabaena* sp.)
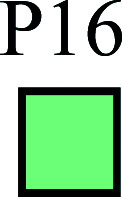	Chlorophytes (*Crucigenia* sp., *Ankistrodesmus* sp., *Selenastrum* sp.), Cyanoprokaryotes (*Spirulina* sp., *Synechococcus* sp., *Planktolyngbya* sp., *Merismopedia* sp.)
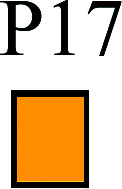	Chlorophytes (*Tetrastrum* sp., *Chlorella* sp., *Ankistrodesmus* sp., *Selenestrum* sp.)
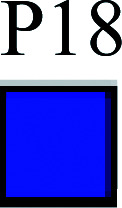	Chlorophytes (*Chlorococcum* sp., *Crucigenia* sp., *Chlorella* sp., *Tetrastrum* sp., *Selenastrum* sp), Cyanoprokaryotes (*Spirulin*a sp., *Synechococcus* sp., *Merismopedia* sp.)
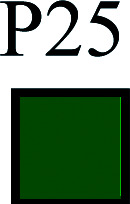	Cyanoprokaryotes (*Spirulina* sp., *Synechococcus* sp.)
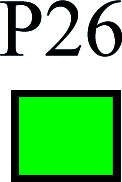	Cyanoprokaryotes (*Synechococcus* sp., *Merismopedia* sp., *Gomphosphaeria* sp.)
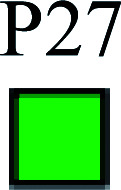	Chlorophytes (*Scenedesmus* sp., *Kirchneriella* sp., *Crucigenia* sp., *Chlamydomonas* sp.), Cyanoprokaryotes (*Spirulina* sp., *Synechococcus* sp., *Synechocystis* sp., *Chroococcus* sp., *Merismopedia* sp.)

**Fig. 8:**
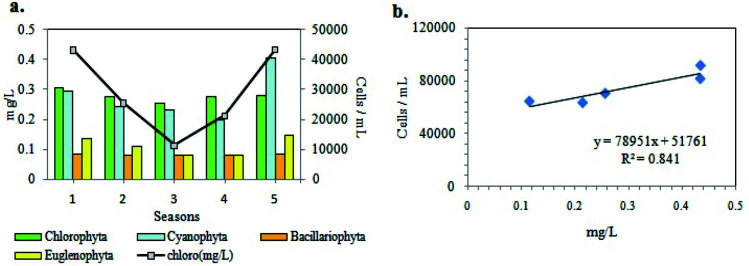
a) Bar graph and (b) scatter plot showing seasonal variations of algal populations (cell count) *vs.* chl content of phytoplankton assemblages. (seasons: 1 = pre-summer, 2 = summer, 3 = monsoon, 4 = post-monsoon, 5 = winter).

The comparison between the seasonal variations of total cell count and chl content of the recorded planktonic algal phyla evidenced that there were almost similar seasonal fluctuations in total chl content and cell count of individual groups (Fig. 8a). A positive relation between total cell count and total chl content was established (Fig. 8b), indicating the phytoplankton's contribution to the chlorophyll concentration of the present habitat water.

### Species diversity index

**Fig. 9:**
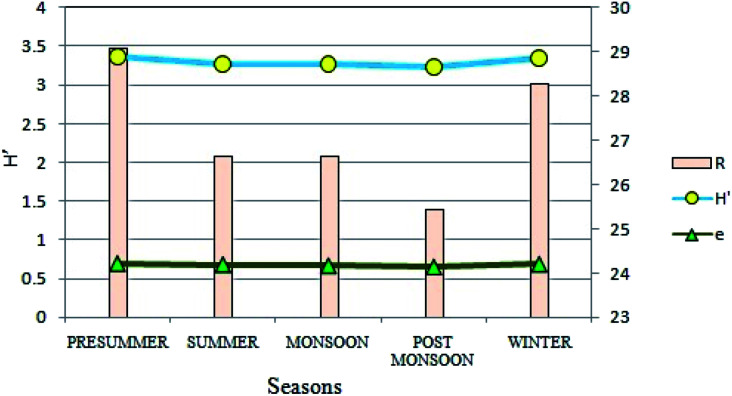
Seasonal variations in Shannon–Wiener's Index (H′), species richness (R) and species evenness (e).

Diversity of phytoplankton population at the present site showed distinct variations on a seasonal basis in terms of different biotic indices (Fig. 9). After FACS based cell counting method, the diversity was measured by Shannon–Wiener's Index (H′)^47^ which varied from 3.23 to 3.37, suggesting an intermediate diversity range as compared to other aquatic habitats like streams and lakes. Highest values were recorded during the pre-summer (H′ = 3.37) and least during the post-monsoon (H′ = 3.23) seasons. Likewise, seasonal variations were evident for species richness as well. Seasonally, species richness was lowest in post-monsoon (25.45) whereas it was highest in the winters (28.29). Species evenness (e), a measure of the contribution of individual taxa to the phytoplankton population showed insignificant seasonal variation (Fig. 9).

### Statistical analysis

**Fig. 10:**
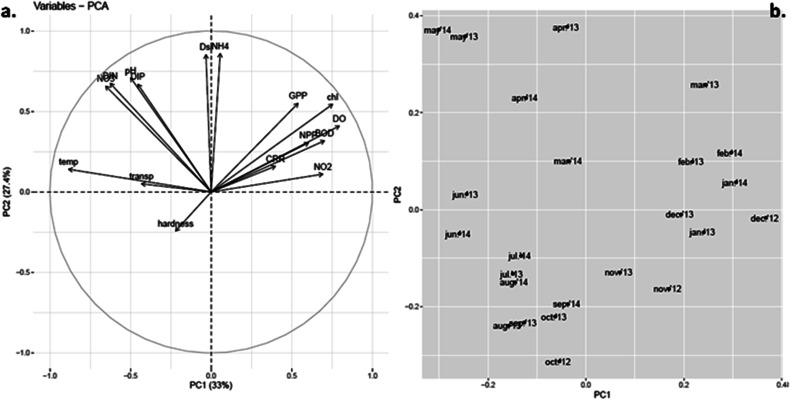
Principal component analysis (PCA) plots of PC1 *vs.* PC2. (a) Loadings plot for environmental variables. (b) Scores plot for sampled months.

In an attempt to aid data interpretation, inferential statistics like Principal Component Analysis (PCA) were used to envisage the possible relationships between biotic and abiotic variables and group these variables on the basis of similarity (Fig. 10). Principal components with eigen values greater than 1 (Kaiser Guttman criterion) were taken into consideration. PCA among environment and biotic variables extracted four significant factors (eigen values greater than 1). PC1 and 2 jointly contributed to 60.4% of the variation within the data. The distribution of the observations along the axes reflects their respective correlations with the variables. Other ordination approaches yielded similar relations between variables (ESI Fig. 1 and 2†).

Among the variables, temperature has the largest but most negative loading for PC1 and thus its variability is explained almost totally by PC1, which accounted for 33% of the total variance (Fig. 10a). The variable chl has equivalent but positive loadings for PC1. This confirmed that chlorophyll content was strongly correlated (negatively) to water temperature. Almost similar length of the vectors for other variables like DO, productivity (GPP, NPP), BOD and nutrients, like NO_2_^–^, in the first quadrant along PC1 showed significant positive correlation with chl and thus in turn negative correlation with water temperature, which was already confirmed from the correlation study among environmental variables (Table 1). High loading values of ammonium nitrogen (NH_4_^+^), Dsi, and moderate values for nitrate (NO_3_^−^), total dissolved inorganic nitrogen (DIN) and dissolved inorganic phosphate (DIP) occurred along PC2 that represented 27.4% of the variance. Thus, these components in the loading plot largely corresponded to high nutrient condition, a possible indication towards eutrophic nature of the habitat. DO and BOD showed high factor loadings along PC1 and intermediate loadings along PC2, which not only indicated similar patterns of variance but established the interdependence between them. The hardness of the water appeared to negatively relate with chl as well. Again, length and direction of the arrows of NO_3_^−^, DIN, DIP and pH suggest their similar pattern of seasonal variation. This was already confirmed from correlation study as well. The scores for the 24 sample months pointed towards the variables driving phytoplankton abundance in each season (Fig. 10b). While the winter and presummer months (Nov-Mar) showed high scores along PC1, summer (Apr-Jun) showed high scores along PC2 and monsoon (Jul-Oct) showed low scores along both axes. Comparing with the loadings of different variables, it was inferred that high phytoplankton abundance in winter and presummer was encouraged by low water temperature and led to high GPP, low nutrient (DIN and DIP) status, high BOD, high DO and low transparency. In summer, high temperature led to waning of phytoplankton bloom and accumulation of nutrients (eutrophication) as indicated by lower GPP, chl, DO and BOD but higher DIN and DIP. Arrival of monsoon diluted the nutrient concentrations, raised transparency but hindered phytoplankton bloom.

**Fig. 11:**
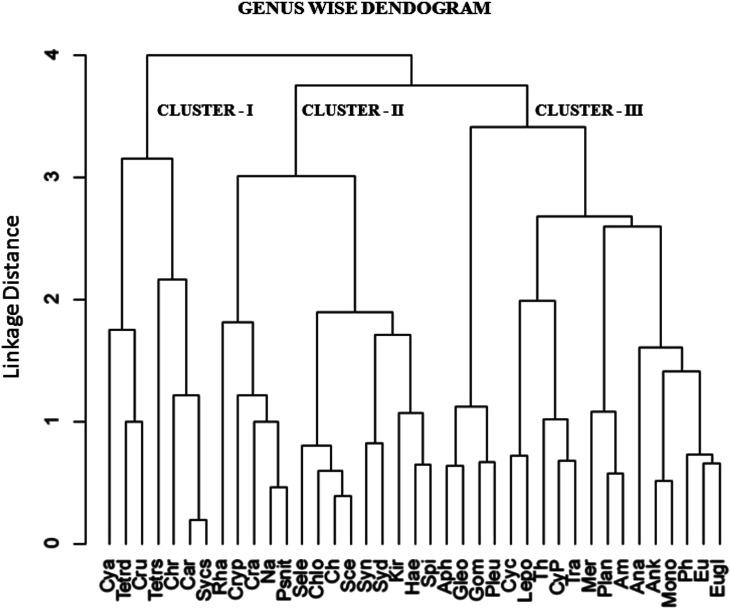
Cluster analysis (CA) of recorded genera using UPGA method (full forms of abbreviated names are listed above).

**Fig. 12:**
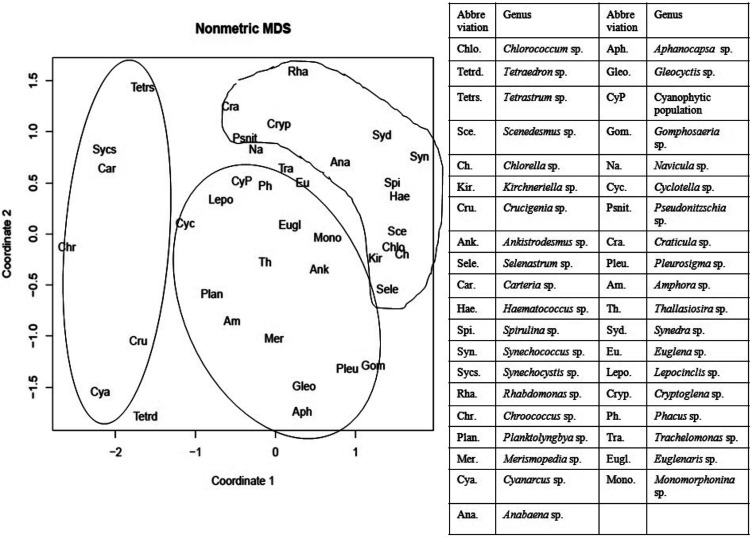
Non-metric multidimensional scaling (NMDS) of different recorded genera considering coordinates 1 and 2. (Inbox: table showing the list of abbreviated genera used for NMDS).

The cluster analysis (CA) (Fig. 11) and NMDS (Fig. 12) ordinations provided a better insight into the seasonal pattern of species based upon their abundance data. The NMDS algorithm ranks distances between objects, and uses these ranks to map the objects nonlinearly onto a simplified, two-dimensional ordination space. From both CA and NMDS plots it was evident that the phytoplankton assemblages could be clustered into three different groups *viz*. Cluster I, II and III. Cluster I comprised of monsoon and post-monsoon dominating population. These included *Cyanarcus* sp. (Cya), *Chroocococcus* sp. (Ch), *Synechocystis* sp. (Sycs), *Tetraedron* sp. (Tetrad), *etc.* Cluster II represented populations which showed higher abundance in pre-summer followed by winter, summer, monsoon and the least in post-monsoon. Cluster III included the winter predominating phytoplankton groups. The Table 4 includes the genera grouped into Cluster I, II and III. Thus from the above two plots it was evident that Euglenophytes were abundant mostly in winters followed by presummer while the Cyanoprokaryotes dominated in the winter season. The Chlorophytes' abundance was maximum during the pre-summer. The maximum chlorophyll content was recorded previously during this season. This could in turn indicate that Chlorophytes accounted for maximum chlorophyll content and phytoplankton productivity.

**Table 4:** List of recorded genera along with their abbreviated names clustered on the basis of cluster analysis

Cluster	Cyanoprokaryota	Chlorophyta	Bacillariophyta	Euglenophyta
Cluster I	(*Cyanarcus* sp.) Cya., (*Chroococcus* sp.) Chr., (*Synechocystis* sp.) Sycs*.*	(*Tetraedron* sp.) Tetra., (*Crucigenia* sp.) Cru., (*Tetrastrum* sp.) Tetras., (*Carteria* sp.) Car.	—	—
Cluster II	(*Spirulina* sp.) Spi., (*Synechococcus* sp.) Syn., (*Rhabdomonas* sp.) Rha.	(*Haematococcus* sp.) Hae., (*Kirchneriella* sp.) Kirch., (*Selenastrum* sp.) Sele*.*, (*Chlorococcum* sp.) Chlo., (*Scenedesmus* sp.) Sce., (*Chlorella* sp.) Ch.	(*Craticula* sp.) Cra., (*Synedra* sp.) Syd., (*Navicula* sp.) Na., (*Psuedonitzschia* sp.) Psnit.	(*Cryptoglena* sp.) Cryp.
Cluster III	(*Aphanocapsa* sp*.*) Aph., (*Gleocystis* sp.) Gleo., (*Gomphospaeria* sp.) Gom., (*Merismopedia* sp.) Mer., (*Anabaena* sp.) Ana., (*Planktolyngbya* sp.) Plan., (Cyanophytic population) Cyp.	(*Ankistrodesmus* sp.) Ank.	(*Pleurosigma* sp.) Pleu., (*Cyclotella* sp.) Cyc., (*Amphora* sp.) Am., (*Thallasiosira* sp.) Th.	(*Lepocinclis* sp.) Lepo., (*Monomorphina* sp.) Mono., (*Phacus* sp.) Ph., (*Euglena* sp.) Eu., (*Euglenaria* sp.) Eugl., (*Trachelomonas* sp.) Tra.

## Discussion

Wetlands being dynamic ecosystems continually undergo natural changes due to infiltration of sediments and nutrients that significantly influence the phytoplankton assemblages. The EKW is renowned for carrying out sewage treatment in conjunction with aquaculture. The water body under investigation, Captain Bhery, had been found to be associated with sewage treatment facility experiencing high flushing rates.^31,34^ It was noted that the environmental variables recorded at Captain Bhery nearly covered the value ranges of corresponding parameters obtained at other different EKW sites.^50–52^ The Captain Bhery was thus considered to be sufficiently stable and dynamic to be a representative water body for the EKW, and the complex interplay of the different hydrological factors and their impact on the phytoplankton population observed at this site could be extended for majority of the EKW. Here the phytoplankton biomass was found to fluctuate seasonally and appeared to be highly influenced by the nutrient regime as well as environmental conditions. The average phytoplankton abundance during the entire study period was found to be 3.7 × 10^5^ cells per mL with maximum abundance during pre-summer and minimum during monsoon. The dominant taxa recorded amongst the entire phytoplankton community were Scenedesmus spp. followed by Merismopedia spp. of Cyanoprokaryota. The FACS study was found to quite helpful in calculating phytoplankton cell count efficiently. Eminent researchers like Crosbie *et al.*,^53^ Tijdens *et al.*,^54^ Toepel *et al.*,^55^ applied FACS to study the phytoplankton community especially in freshwater environment. Cellamare *et al.*,^56^ also sorted 175 algal taxa from different freshwater ecosystems including seasonal dynamics of Synechococcus spp using FACS method.

The division Cyanoprokaryota and Euglenophyta mainly predominated during the winter seasons. The CA demonstrated progressive change of dominance through a warmer to cooler temperature gradient. The chlorophyll content and the phytoplankton count including FACS observations suggested a seasonal trend in phytoplankton assemblages with monsoon being least abundant due to seasonal precipitation, and presummer being the maximum. Similarities between variation patterns of total chlorophyll content and phytoplankton cell count suggested that the autotrophic productivity of the present ecosystem was primarily regulated by the phytoplankton biomass as indicated from GPP value also. It has already been observed that the chlorophyll values accorded with that of eutrophic ecosystem.^57^ The pH recorded was slightly alkaline which, from the above results, was evidently contributed by the different nutrients present therein. Besides, the higher pH values obtained validated the occurrence of eutrophication.^58,59^ An important ecological factor regulating phytoplankton growth is water temperature.^32^ Temperature seemed to be primarily responsible for the shifts in phytoplankton assemblages as significant negative correlation of temperature with chlorophyll, DO, GPP and NPP was obtained from the study. This inference was supported by PCA results, which indicated that water temperature and annual precipitation were major determinants of phytoplankton abundance, which was highest in winter and lowest in monsoon (Fig. 8 and 10). Although chlorophyll shows strong negative correlation with temperature, it is not winter (when temperature is minimum that productivity peaks. It is in presummer when the mix of high nutrient levels and optimum temperature causes maximium productivity. Both abundance and diversity of phytoplankton taxa diminished from presummer to summer despite eutrophic conditions in summer (Fig. 3–5, 8 and 9). Mesotrophic status was achieved in winter and presummer by assimilation of excess nutrients by phytoplankton, which also raised GPP and DO by oxygenic photosynthesis, and in turn encouraged high microbial abundance as indicated by high BOD and CRR.

The DO is essential to all forms of aquatic life, including those organisms responsible for the self-purification processes in natural waters.^59^ A regulatory network of DO along with photosynthetic activity and primary productivity therein equilibrates the ecological balance of the ecosystem. The present findings accorded with the previous reports of Hardy^60^ where DO shows a positive correlation with the phytoplankton biomass (Table 1). According to Ganf and Horne,^61^ if in a productive aquatic ecosystem respiration accounted for large proportion of GPP, it would be a measure of eutrophic nature. In general, decomposition of the sewage, dead plankton *etc.* along with respiration of the inhabitants are responsible for creating avenues for high CRR and ultimately BOD of the water column. The present investigation recorded higher BOD levels, which probably emphasized on the eutrophic status of the selected site. A positive correlation obtained between DO and BOD is indicative of higher heterotrophic microbial community along with planktonic autotrophs supported by higher CRR values and lower percentages of oxygen saturation. However, the obtained DO values ranged up to of 8.14 mg L^−1^ which according to WHO,^60^ supported the survival of biological communities including fish production. Higher values of GPP and its significant positive correlation with DO were useful in supporting the fact that phytoplankton contributes a natural method of biological purification for the sewage treatment in EKW. This was further supported by other studies from other EKW sites such as Dasgupta *et al.*^62^

During sewage treatment the microbial degradation of the sewage releases the nutrients stored in it, creating eutrophic conditions, which in turn support high rates of primary productivity.^32,35^ The excretion of nitrogenous compounds by fish is also a source of NO_3_^−^, NO_2_^−^, NH_4_^+^and other inorganic substances.^63^ Major nutrient like nitrogen occurs in natural waters in various forms, including NO_3_^−^, NO_2_^−^ and NH_4_^+^. The NO_3_^−^ is the essential nutrient for many photosynthetic autotrophs and has been identified as the growth limiting nutrient.^59^ However, in municipal and industrial waste-waters or effluents including biological treatment plants, NO_3_^−^ concentrations are enhanced resulting in eutrophication.^57^ Similarly NO_2_^−^ concentrations higher than 21.74 μM and NH_4_^+^ greater than 11.11 μM could be an indication of organic pollution.^59^ High availability of NO_3_^−^ in EKW not only encourages phytoplankton abundance but also raises anaerobic metabolism, particularly under oxic conditions, while NO_2_^−^ plays only a transient role in N cycling.^64^ Another major nutrient of aquatic systems for phytoplankton development is phosphate mostly in form of DIP which in general ranges from 0.053 to 0.21 μM in most natural surface waters.^57^ The present records also showed the positive role of NO_3_^−^, NH_4_^+^ and DIP on phytoplankton biomass growth.^65^ However, loadings of water parameters, particularly temperature, transparency, chlorophyll content, BOD and DO, obtained by PCA showed that biomass-induced opacity of the water column was mainly caused by photosynthetic microorganisms, indicating a productive ecosystem occurring in winter and presummer (Fig. 7–10). Since this productivity was seasonal, an effective way of sewage treatment would be periodic harvesting of the nutrient-containing photosynthetic biomass from the littoral zone throughout winter and presummer, leading to an oligotrophic and oxygenated habitat for pisciculture.

## Conclusion

In summary, it may be said that the EKW station is suitable for phytoplankton population growth. Elevated nutrient levels, particularly species of nitrogen and to a lesser extent DIP, contribute to the growth of phytoplankton resulting in high levels of DO, GPP and NPP. These in turn favor the purification process, provided that the photosynthetic biomass is removed at the end of the productive season to prevent decay and anoxia. The levels of phytoplankton biomass, pH, BOD, major nutrient concentrations of the water body and the heterotrophic microbial respiration were in support for the wetland under study to be eutrophic. Even though BOD levels are high, significantly higher DO along with elevated levels of GPP and NPP support the growth of fish making it suitable for aquaculture. FACS based phytoplankton community study was found to be useful to account for their diversity and abundance. Seasonal variation in the phytoplankton standing stock was primarily regulated by environmental variables like nutrient availability, temperature variations and annual precipitation. From the total cell count of individual phytoplankton genus, it could be inferred that Scenedesmus sp. (Chlorophyta) followed by Merismopedia sp. (Cyanoprokaryota) were most abundant.

## Conflicts of interest

There are no conflicts to declare.

## Supplementary Material

RA-008-C7RA12761H-s001
