# Correction: Phytoplankton nutrient dynamics and flow cytometry based population study of a eutrophic wetland habitat in eastern India, a Ramsar site

**DOI:** 10.1039/d4ra90087a

**Published:** 2024-08-14

**Authors:** Anindita Singha Roy, Prakash Chandra Gorain, Ishita Paul, Sarban Sengupta, Pronoy Kanti Mondal, Ruma Pal

**Affiliations:** a Phycology Laboratory, Department of Botany, University of Calcutta 35, Ballygunge Circular Road Kolkata 700019 West Bengal India rpalcu@rediffmail.com +91-9433116320; b Agricultural and Food Engineering Department, Indian Institute of Technology Kharagpur Kharagpur 721 302 India; c Human Genetics Unit, Indian Statistical Institute Kolkata 700108 West Bengal India

## Abstract

Correction for ‘Phytoplankton nutrient dynamics and flow cytometry based population study of a eutrophic wetland habitat in eastern India, a Ramsar site’ by Anindita Singha Roy *et al.*, *RSC Adv.*, 2018, **8**, 9530–9545, https://doi.org/10.1039/C7RA12761H.

The authors regret that there was an error in the figure caption for Fig. 2. The corrected figure caption is below.

**Fig. 2:**
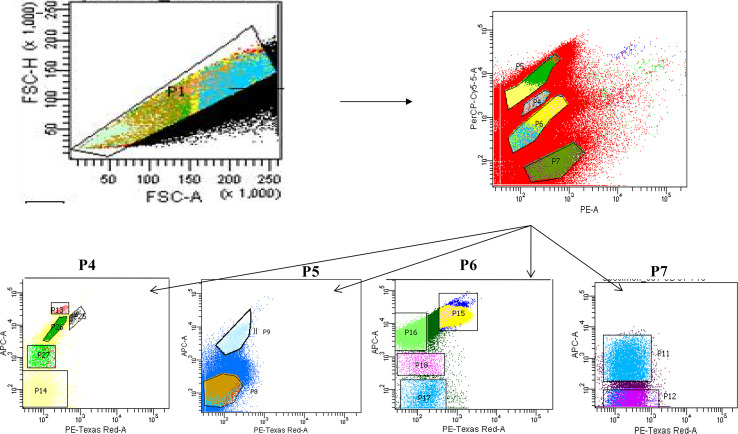
Bivariate scatter plots analyzed using FACSort flow cytometry, showing gating of phytoplankton population based on pigment auto-fluorescence and their cell size. P7 refers to the summer data also presented in Fig. 7.

The Royal Society of Chemistry apologises for these errors and any consequent inconvenience to authors and readers.

