# Supplementary material for: Phytoplankton nutrient dynamics and flow cytometry based population study of a eutrophic wetland habitat in eastern India, a Ramsar site
Source: RSC Adv. 2018 Mar 5;8(17):9530–45. doi: 10.1039/c7ra12761h (PMC9078691; doi:10.1039/c7ra12761h)
Supplement: RA-008-C7RA12761H-s001 [file RA-008-C7RA12761H-s001.pdf]

Electronic Supplementary Material (ESI) for RSC Advances.
This journal is © The Royal Society of Chemistry 2018

Supplementary figures:

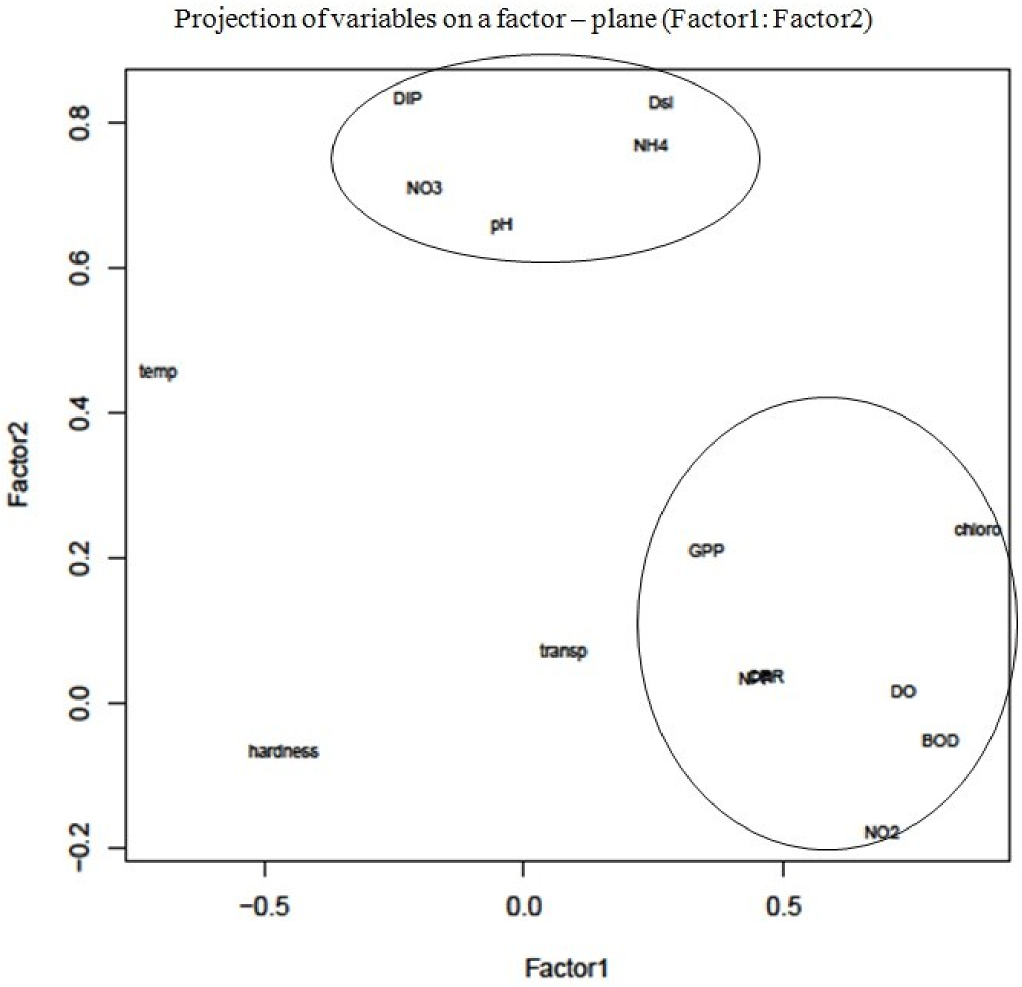

Supplementary figure 1: Factor analysis (FA) plot of factor 1 vs. factor 2 for environmental variables recorded from the study area during the entire study period

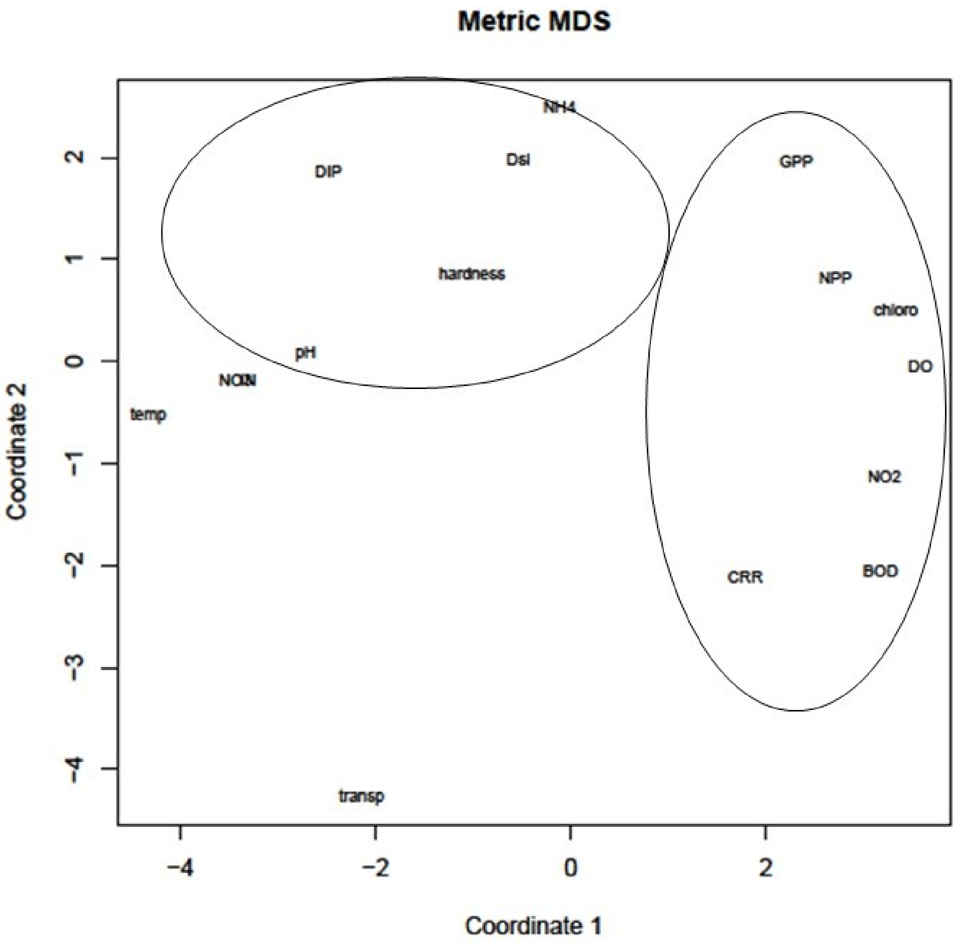

Supplementary figure 2: Metric multidimensional scaling (MMDS) plot of Coordinate 1 vs. Coordinate 2 for environmental variables recorded from the study area during the entire study period
